# In vitro and in vivo cytotoxicity of troglitazone in pancreatic cancer

**DOI:** 10.1186/s13046-017-0557-6

**Published:** 2017-07-03

**Authors:** Megumi Fujita, Ai Hasegawa, Motohiro Yamamori, Noboru Okamura

**Affiliations:** Department of Clinical Pharmacy, School of Pharmacy and Pharmaceutical Sciences, Mukogawa Women’s University, 11-68 Koshien-kyuban-cho, Nishinomiya, Hyogo 663-8179 Japan

**Keywords:** Troglitazone, PPARγ, Pancreatic cancer, Apoptosis, JNK MAPK, In vivo

## Abstract

**Background:**

Troglitazone (TGZ) is a peroxisome proliferator-activated receptor gamma (PPARγ) agonist that has been investigated as a potential chemopreventive and chemotherapeutic agent. However, the antitumor efficacy and mechanisms of TGZ in pancreatic cancer have not been extensively investigated. This study was performed to investigate the in vitro and in vivo effects of TGZ against pancreatic cancer cell lines, as well as its action mechanisms in terms of PPARγ dependency and the Akt and mitogen-activated protein kinase (MAPK) pathways. We also evaluated the effects of TGZ on cell invasion and migration.

**Methods:**

MIA Paca2 and PANC-1 human pancreatic cancer cell lines were used. Cell viability and caspase-3 activity were detected using fluorescent reagents, and chromatin condensation was observed after staining the cells with Hoechst 33342. Protein expression levels were detected by western blot analysis. Invasion and migration assays were performed using 24-well chambers. The in vivo antitumor effects of TGZ were investigated in nude mice inoculated with MIA Paca2 cells. Mice were orally administered TGZ (200 mg/kg) every day for 5 weeks, and tumor volumes were measured bi-dimensionally.

**Results:**

TGZ showed dose-dependent cytotoxicity against both cell lines, which was not attenuated by a PPARγ inhibitor. Further, TGZ induced chromatin condensation, elevated caspase-3 activity, and increased Bax/Bcl-2 relative expression in MIA Paca2 cells. TGZ also increased phosphorylation of Akt and MAPK (ERK/p38/JNK) in both cell lines, and a JNK inhibitor significantly increased the viability of MIA Paca2 cells. TGZ moderately inhibited cell migration. Tumor growth in the MIA Paca2 xenograft model was inhibited by TGZ administration, while mouse body weights in the treated group were not different from those of the vehicle administration group.

**Conclusion:**

We demonstrated for the first time the in vivo antitumor effects of TGZ in pancreatic cancer without marked adverse effects. TGZ induced mitochondria-mediated apoptosis in MIA Paca2 cells, and its cytotoxic effects were PPARγ-independent and occurred via the JNK pathway. Our results indicate that TGZ is a potential approach for the treatment of pancreatic cancer and warrants further studies regarding its detailed mechanisms and clinical efficacy.

## Background

Pancreatic cancer is the fourth leading cause of cancer-related deaths in Japan [1]. Although pancreatectomy is the most effective treatment at early stages, advanced pancreatic cancer is relatively resistant to chemotherapy and radiotherapy, and is still associated with a poor prognosis with a five-year survival rate of 7% in Japan for all five disease stages [1]. Gemcitabine has been the standard therapy for advanced pancreatic cancer for over a decade [2]. Recently, monotherapy with S-1, an oral 5-fluorouracil prodrug (tegafur) combined with two modulators (5-chloro-2, 4-dihydroxypyridine and potassium oxonate), was reported to extend both overall and relapse-free survival compared to that reported with gemcitabine [3]. Furthermore, additional novel combination therapies have been developed, such as gemcitabine plus erlotinib; a combination of leucovorin, 5-fluorouracil, irinotecan, and oxaliplatin (FOLFIRINOX); and gemcitabine plus nab-paclitaxel. Although overall survival has been significantly prolonged with these regimens, the effect remains insufficient, and it is therefore necessary to develop a novel effective strategy for pancreatic cancer treatment.

Peroxisome proliferator-activated receptor gamma (PPARγ) belongs to the nuclear hormone receptor transcription factor superfamily, playing major roles in adipogenesis, glucose metabolism, and angiogenesis [4]. PPARγ is expressed in a variety of normal tissues and tumor sites [5–7], as well as in pancreatic cancer [8, 9]. While the role of PPARγ in tumor sites remains poorly defined, the thiazolidinedione family of PPARγ agonists, such as troglitazone (TGZ), pioglitazone (PGZ) and rosiglitazone, have been shown to regulate growth and survival in a number of cancer cell lines [10], suggesting that TGZ may serve as an effective approach for the treatment of pancreatic cancer. TGZ has been reported to induce antitumor effects via multiple signaling mechanisms, but mainly in a PPARγ-independent manner [10, 11]. However, there have been few reports regarding the antitumor efficacy and mechanisms of TGZ in pancreatic cancer [8, 9]. Moreover, there are no reports concerning PPARγ dependency and in vivo antitumor efficacy of TGZ in pancreatic cancer cells, although PGZ was evaluated using an in vivo model [9].

In the present study, we investigated whether the PPARγ agonist, TGZ, exhibited in vitro cytotoxicity against two human pancreatic cancer cell lines, and clarified its mechanisms in terms of PPARγ dependency, apoptosis, and the mitogen-activated protein kinase (MAPK) pathway. Furthermore, we examined the effects of TGZ on cell invasion and cell migration, as well as in vivo antitumor effects.

## Methods

### Chemicals

TGZ and pioglitazone were purchased from LKT Laboratories (St. Paul, MN) and BioVision (Milpitas, CA), respectively, and were dissolved in dimethyl sulfoxide (DMSO, Nacalai Tesque, Kyoto, Japan) just before use. The final DMSO concentration in the media did not exceed 0.1%. GW9662 (PPARγ inhibitor), SP600125 (JNK inhibitor), and SB202190 (p38 inhibitor) were obtained from Merck Millipore (Billerica, MA).

### Cells and cell culture

MIA Paca2 and PANC-1 cells were provided by the RIKEN BRC through the National Bio-Resource Project of the MEXT (Ibaraki, Japan), and were used as human pancreatic cancer cell models. They were cultured in DMEM (Wako Pure Chemical Industries, Osaka, Japan). Media were supplemented with 10% heat-inactivated fetal bovine serum (FBS; GIBCO^®^, Thermo Fisher Scientific, Waltham, MA) and 50 U/mL penicillin-50 μg/mL streptomycin (Nacalai Tesque). Cells were cultured in an atmosphere of 95% air and 5% CO_2_ at 37 °C and were subcultured every 3 or 4 days.

### Cell viability

Cell viability was measured with Cell Quanti-Blue™ (BioAssay Systems, Hayward, CA). Briefly, cells were seeded into 96-well plates (Asahi Glass, Tokyo, Japan) at a density of 1 × 10^4^ cells/well and incubated for 24 h. The cells were treated with TGZ in the presence or absence of other chemicals for a further 24 h using FBS-free medium. The assay utilizes the conversion of alamar blue reagent to fluorescent resorufin by metabolically active cells. The resorufin signal was measured in a CytoFluor^®^ Series 4000 Fluorescence Multi-Well Plate Reader (PerSeptive Biosystems, Framingham, MA) at an excitation wavelength of 530 nm and an emission wavelength of 580 nm. The 50% growth inhibitory concentrations (IC_50_) were calculated according to the sigmoid inhibitory effect model *E* = IC_50_^γ^/(IC_50_^γ^ + C^γ^), where *E* represents the surviving fraction (% of control), C represents the drug concentration in the medium, and γ represents the Hill coefficient. For co-exposure studies, the TGZ dosage was set to approximately the IC_50_ value for each cell line.

### Detection of chromatin condensation (fluorescence microscopy)

For nuclei staining, cells were treated with TGZ for 24 h at the IC_50_ concentrations for each cell line. Immediately after treatment, the nuclear chromatin of trypsinized cells was stained with 80 μg/mL Hoechst 33342 (Nacalai Tesque) in the dark at 20 °C for 15 min. They were then observed with a brightfield fluorescence microscope (VANOX; Olympus, Tokyo, Japan) under UV excitation. Cells with condensed chromatin were photographed at 40-fold magnification. In addition, at 20-fold magnification, more than 100 cells with condensed chromatin were counted in each experiment, and their percentage of the population was calculated.

### Antibodies

Rabbit monoclonal antibodies against PPARγ (81B8), Bax, Bcl-2, phospho-Akt (Ser473; D9E), and Akt (C67E7), phospho-ERK (Thr202/Tyr204; D31.14.4E), ERK (137 F5), phospho-JNK (Thr183/Tyr185; 81E11), JNK (56G8), phospho-p38 (Thr180/Tyr182; D3F9), and p38 (D13E1) were purchased from Cell Signaling Technology (Danvers, MA). Mouse monoclonal antibody against β-actin (C4) was from Santa Cruz Biotechnology (Dallas, TX). Horseradish peroxidase-linked goat anti-rabbit IgG was obtained from Santa Cruz Biotechnology and sheep anti-mouse IgG was obtained from GE Healthcare (Buckinghamshire, UK).

### Western blot analysis

Cells (1.75 × 10^6^) were plated in 100-mm dishes 24 h before treatment and then treated with TGZ (50 μM) for 1, 4, 8, or 24 h. Cells were washed with ice-cold phosphate-buffered saline (PBS), harvested by scraping, and centrifuged at 300 × *g* and 4 °C for 5 min. Lysis buffer (20 mM Tris (pH 7.5), 150 mM NaCl, 1% Triton™ X-100, 0.5% sodium deoxycholate, 1 mM EDTA, 0.1% SDS, 1 mM NaF, 1 mM Na_3_VO_4_, and 0.1% protease inhibitor cocktail (Merck Millipore)) was added to pellets, and then cells were sonicated briefly, followed by incubation on ice for 20 min. Cell extracts were centrifuged at 16,000 × *g* and 4 °C for 15 min, and supernatants were transferred to new tubes. Protein concentrations were determined by BCA protein assays. The samples were mixed with the same volume of 2× SDS-PAGE sample buffer containing β-mercaptoethanol (Nacalai Tesque) followed by boiling for 5 min, and proteins (15 μg/lane) were loaded onto 10% SDS-polyacrylamide gels. After electrophoresis, the proteins were transferred to a polyvinylidene difluoride membrane (GE Healthcare) and blocked with Tris-buffered saline-0.1% Tween^®^ 20 (TBS-T) containing 2% ECL Advance™ Blocking Agent (GE Healthcare) for 1 h. Blocked membranes were reacted with primary antibodies (diluted 1:10,000) for 1 h at 20 °C followed by five washes with TBS-T. After incubation with the secondary antibody (diluted 1:25,000) for 1 h at 20 °C, membranes were washed five times. Signal was visualized using ECL Advance™ detection reagents (GE Healthcare). Expression levels were analyzed using Image J software.

### Fluorometric assay of caspase-3 activity

Caspase-3 activity was assessed using a Fluorometric Caspase 3 Assay Kit (Merck Millipore) according to the manufacturer’s instructions. Briefly, cells were seeded in 24-well plates at a density of 6 × 10^4^ cells/well followed by 24 h incubation. After exposure to TGZ for 8 h, the cells were harvested with lysis buffer (50 mM HEPES, pH 7.4, 5 mM CHAPS, and 5 mM DTT). The reaction buffer, including 16.6 μM Acetyl-Asp-Glu-Val-Asp-7-amido-4-methylcoumarin (Ac-DEVD-AMC), a caspase-3-specific substrate, was added to the wells, and the production of AMC was sequentially detected in a CytoFluor^®^ Plate Reader at an excitation wavelength of 360 nm and an emission wavelength of 460 nm. Enzyme activities were determined as initial velocities expressed as nmol AMC/min/mL, which were then corrected using the protein concentration in each well as determined by the BCA protein assay (Thermo Fisher Scientific).

### Invasion and migration assay

An invasion assay was performed using 24-well BD BioCoat™ Matrigel^®^ invasion chambers with 8.0-μm polycarbonate membrane filters (Corning; Corning, NY). Cells were seeded on membranes at a density of 2 × 10^5^ cells/well with FBS-free medium; the membranes were placed into the lower chambers and 10% FBS-containing medium was added. FBS was used as a chemoattractant. After being cultured with or without TGZ (maximum concentration without cytotoxicity: 10 μM and 1 μM in MIA Paca2 and PANC-1 cell lines, respectively) for 24 h, cells on the upper surface of the membranes were removed using a cotton swab. Invasive cells that had penetrated through the membrane pores and migrated to the underside of the membranes were stained with Giemsa solution after fixation with 100% methanol. Cell numbers were quantified under a microscope, and the average cell number determined from quantification of four locations. The migration assay used the same protocol as the invasion assay except that non-coated chambers were used.

### Animal model

Animal experiments were performed in accordance with the guidelines of the Institutional Animal Use Committee of Mukogawa Women’s University, and the procedure was approved by the Committee. Balb/c male mice (4 weeks old) were purchased from Japan SLC (Shizuoka, Japan) and subcutaneously inoculated in the back with MIA Paca2 cells (5 × 10^6^ cells/100 μL in PBS) 14 days prior to starting TGZ administration. Mice were then orally administered 200 mg/kg TGZ in 0.5% methylcellulose solution (Nacalai Tesque) or vehicle daily for 5 weeks. Tumor size was measured bi-dimensionally and the volume was calculated using the formula (length × width^2^) × 0.5. Body weights of mice were also monitored throughout the experiment.

### Statistical analysis

The data are expressed as the mean +/- the SD. Statistical significance was examined using Student’s or Welch’s *t*-test for comparing two groups. Non-repeated one-way analysis of variance (ANOVA) followed by Dunnett’s post-hoc test was used for multiple comparisons. *P* < 0.05 was considered statistically significant.

## Results

### Effect of TGZ and PGZ on cell viability

**Fig. 1:**
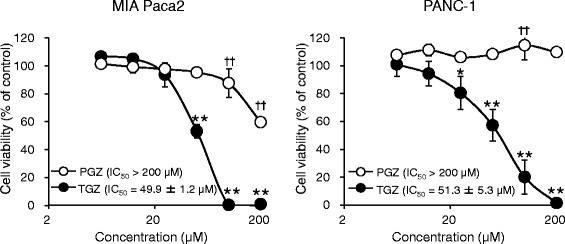
Cytotoxic effects of troglitazone (TGZ) and pioglitazone (PGZ) in two pancreatic cancer cell lines. MIA Paca2 or PANC-1 cells were pre-cultured for 24 h at a density of 1 × 10^4^ cells/well in 96-well plates and treated with TGZ or PGZ for 24 h. Cell viability was assessed by fluorescence assay, and data represent the mean ± SD from four independent preparations. Statistical significance was assessed by Dunnett’s test (control vs. each concentration of TGZ). * *p* < 0.05 and ** *p* < 0.01 vs. control (for TGZ). † *p* < 0.05 and †† *p* < 0.01 vs. control (for PGZ)

Figure 1 shows the effects of TGZ and PGZ on the viability of the two pancreatic cancer cell lines. PGZ is another thiazolidinedione antidiabetic drug, and was used for comparison with TGZ. TGZ showed dose-dependent cytotoxicity with IC_50_ values of 49.9 ± 1.2 and 51.3 ± 5.3 μM in MIA Paca2 and PANC-1 cells, respectively, whereas the IC_50_ values for PGZ were higher than 200 μM.

### Involvement of PPAR in effects of TGZ on cell viability

**Fig. 2:**
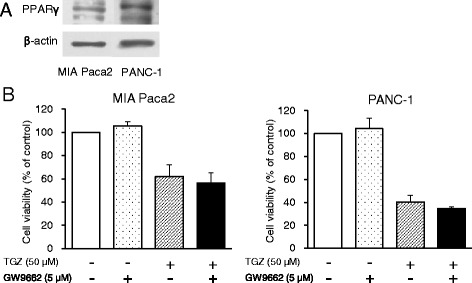
PPARγ protein expression and the effects of the PPARγ inhibitor GW9662 on TGZ-induced cell death. **a** Cells (1.75 × 10^6^) were incubated for 24 h, and extracted protein (15 μg) was analyzed by western blot for PPARγ expression. **b** Cells were pre-cultured for 24 h at a density of 1 × 10^4^ cells/well in 96-well plates and exposed to TGZ (50 μM) with or without GW9662 (5 μM) for 24 h. Cell viability was assessed by fluorescence assay (*n* = 4–6). Statistical significance was assessed by *t*-test (TGZ vs. TGZ + GW9662, n.s., not significant). PPARγ, peroxisome proliferator-activated receptor gamma; TGZ, troglitazone

To investigate the involvement of PPARγ in TGZ-mediated reduction of cell viability, we examined PPARγ protein levels using western blotting and ascertained whether a PPARγ antagonist, GW9662, could recover cell viability. Although PPARγ was detected in both cell lines (Fig. 2a), 5 μM of GW9662 did not increase the viability of TGZ-treated cells (Fig. 2b). We also conducted dose-response studies for GW9662 up to 20 μM, but cell viability was not rescued, and some cytotoxicity was observed when the cells were exposed to GW9662 at high concentrations (data not shown).

### Effect of TGZ on nuclear morphology, caspase-3 activity, and Bax/Bcl-2 expression

**Fig. 3:**
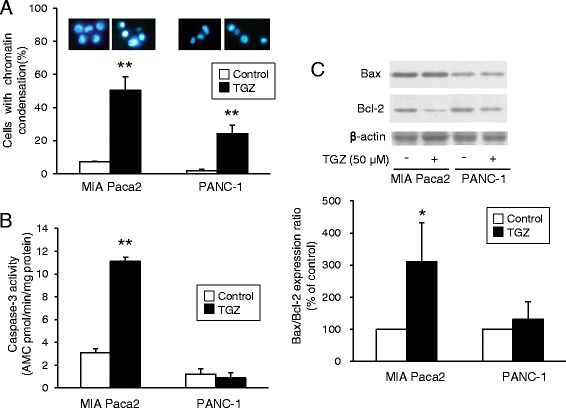
Apoptosis assays for TGZ. **a** Representative fluorescence microscopy images of cells stained with Hoechst 33342 and the percentage of cells with chromatin condensation. Cells were treated with TGZ (50 μM) for 24 h and stained with Hoechst 33342 for 15 min at 20 °C. Cells were then observed using fluorescence microscopy under UV excitation, and the percentage of cells showing chromatin condensation was determined. Data represent the mean + SD from four independent preparations. **b** Effects of TGZ on caspase-3 activity. Cells (6 × 10^4^ cells/well) were seeded in 24-well plates followed by 24 h incubation. After exposure to TGZ (50 μM) for 8 h, caspase-3 activity was assessed using a fluorometric Caspase 3 Assay Kit. Enzyme activities were determined as initial velocities corrected by protein quantity (*n* = 4). **c** Bax and Bcl-2 protein expression. Cells (1.75 × 10^6^) were incubated for 24 h and exposed to TGZ (50 μM) for another 24 h. Extracted protein (15 μg) was analyzed by western blotting. Bcl-2 expression levels were corrected using β-actin and analyzed from three independent preparations. * *p* < 0.05 and ** *p* < 0.01 vs. control (*t*-test). TGZ, troglitazone

Next, we conducted several experiments examining apoptosis. When cells were exposed to TGZ at 50 μM for 24 h, chromatin condensation, a typical morphological change characteristic of apoptosis, was observed after Hoechst 33342 staining. TGZ significantly increased the percentage of MIA Paca2 and PANC-1 cells showing chromatin condensation from 7 to 50% and from 2 to 24%, respectively (Fig. 3a). TGZ also significantly increased the activity of caspase-3, a downstream component of the caspase cascade, by approximately 3.5-fold in MIA Paca2 cells, although no remarkable changes were observed in PANC-1 cells (Fig. 3b). Figure 3c shows the western blot analysis of the pro-apoptotic Bax protein and the anti-apoptotic Bcl-2 protein levels, which are involved in the mitochondrial apoptosis pathway. TGZ markedly decreased Bcl-2 expression by approximately 70% in MIA Paca2 cells and moderately decreased levels in PANC-1 cells. However, Bax expression was not significantly affected in both cell lines.

### Involvement of Akt and MAPK signaling in TGZ-induced cell death

**Fig. 4:**
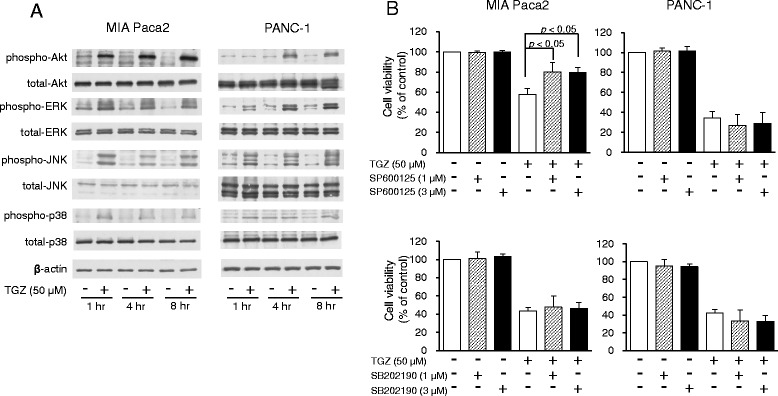
Involvement of Akt and MAPK signaling in TGZ-induced cell death. **a** Akt and MAPK protein expression. Cells (1.75 × 10^6^) were pre-cultured for 24 h in 100-mm dishes and treated with TGZ (50 μM) for various durations. Protein (15 μg) was analyzed by western blot for expression of Akt, ERK, JNK, p38, and the phosphorylated forms of each protein. β-Actin was used as a loading control. **b** Effects of a JNK inhibitor (SP600125) and a p38 inhibitor (SB202190) on TGZ-induced cell death. Cells were pre-cultured for 24 h at density of 1 × 10^4^ cells/well in 96-well plates and then exposed to TGZ (50 μM) in the presence or absence of SP600125 or SB202190 (1 and 3 μM, respectively) for 24 h. Cell viability was assessed by fluorescence assay and is expressed as mean + SD (*n* = 3–5). Statistical significance was assessed by Dunnett’s test (TGZ vs. TGZ + inhibitors, n.s., not significant). MAPK, mitogen-activated protein kinase; ERK, extracellular signal-regulated kinase; JNK, c-Jun N-terminal kinase; TGZ, troglitazone

To examine the involvement of growth, survival, and death pathways, we examined the expression of the Akt and MAPK subfamily proteins: extracellular signal-related kinase (ERK), c-Jun N-terminal kinase (JNK), and p38. TGZ increased the phosphorylation levels of all these proteins in both cell types, although the phospho-p38 effect is not entirely clear with PANC-1 cells (Fig. 4a). As JNK and p38 are known to induce cell death, we also examined whether JNK and p38 inhibitors affected TGZ-induced cytotoxicity. We first confirmed that the phosphorylation of JNK was inhibited by SP600125 at 1 μM (data not shown), and we had previously confirmed that 3 μM of SB202190 completely blocked phosphorylation of p38 [12]. Co-exposure to 1 μM SP600125 significantly elevated the viability of TGZ-treated MIA Paca2 cells, while no significant recovery was observed for PANC-1 cells (Fig. 4b). However, 3 μM SB202190 did not attenuate TGZ cytotoxicity.

### Effects of TGZ on cell invasion and migration

**Fig. 5:**
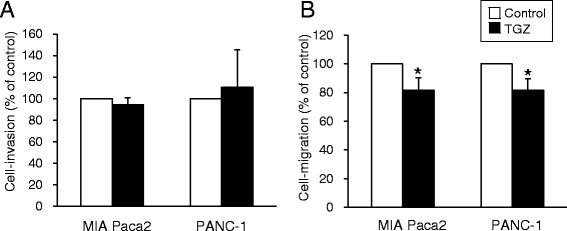
Effects of TGZ on cell invasion and cell migration. **a** The cell invasion assay was performed using 24-well BD BioCoat™ Matrigel® invasion chambers with 8.0-μm polycarbonate membrane filters. Cells were seeded on membranes at 2 × 10^5^ cells/well with FBS-free medium, after which the membranes were placed into the lower chamber and incubated with 10% FBS-containing medium. After culture with or without TGZ (MIA Paca2: 10 μM, PANC-1: 1 μM) for 24 h, cells on the upper surface of the membranes were removed using a cotton swab. Invasive cells that penetrated through the pores and migrated to the underside of the membrane were stained with Giemsa solution after fixation with 100% methanol. Cell number was quantified under microscopy. **b** The cell migration assay was conducted in the same manner as the cell invasion assay, except for the use of non-coated chambers. Data represent the mean + S.D. from three or four independent preparations. * *p* < 0.05 vs. control (*t*-test). TGZ, troglitazone; FBS, fetal bovine serum

TGZ significantly reduced cell migration levels by approximately 20% compared to control migration values, whereas cell invasion levels were not affected (Fig. 5).

### In vivo antitumor effects of TGZ

**Fig. 6:**
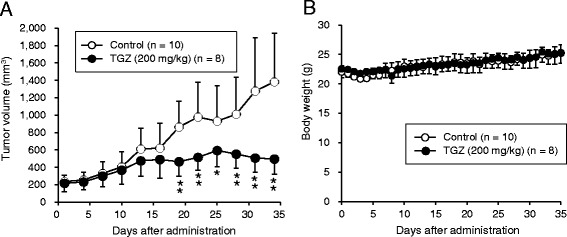
In vivo antitumor effects of TGZ. **a** Tumor volume. Balb/c male mice were subcutaneously inoculated in the back with MIA Paca2 cells (5 × 10^6^ cells) 14 days before the start of TGZ administration. Mice were then orally administered vehicle (control, *n* = 10) or 200 mg/kg of TGZ (*n* = 8) daily for 5 weeks. Tumor volume was measured bi-dimensionally and volume was calculated by the formula (length × width^2^) × 0.5. **b** Mouse body weights were monitored throughout the experiment. Data are presented as mean ± SD. * *p* < 0.05 and ** *p* < 0.01 vs. control on each individual day (*t*-test). TGZ, troglitazone

To clarify the effects of TGZ in controlling tumor growth in vivo, we administered TGZ to mice inoculated with MIA Paca2 cells. TGZ exhibited inhibitory effects on tumor growth in the MIA Paca2 xenograft model (Fig. 6a); however, the body weights of mice were not affected compared to those of the vehicle administration group (Fig. 6b).

## Discussion

Pancreatic cancer has one of the poorest outcomes worldwide. While some effective chemotherapy measures have been reported recently, their efficacy unfortunately remains limited. As PPARγ agonist thiazolidinediones have been shown to regulate growth and survival in a number of cancer cell lines [10], we designed this study to evaluate the antitumor effects of TGZ on pancreatic cancer cells in vitro and in vivo, and investigated its mechanism of cytotoxicity.

TGZ exhibited dose-dependent cytotoxicity with IC_50_ values of approximately 50 μM and was more effective than PGZ was (Fig. 1). Similar to our results, previous reports have shown that TGZ showed higher efficacy than did the other thiazolidinediones [13–15], suggesting that TGZ could be a good antitumor candidate.

To investigate the association of PPARγ with TGZ-induced cytotoxicity, we detected PPARγ protein levels and confirmed the effect of an irreversible PPARγ antagonist, GW9662, on TGZ-induced cell death. Although PPARγ was appreciably detected in both cell lines, GW9662 did not recover cell viability (Fig. 2), suggesting that the TGZ-mediated cytotoxic effect was PPARγ-independent. The anticancer mechanisms of TGZ remain elusive, with the literature reporting both PPARγ-dependent and independent cytotoxicity. For example, PPARγ-dependent TGZ cytotoxicity was reported in lung cancer [16] and osteoblastic cells [17], while PPARγ-independent cytotoxicity was observed in colon cancer [18], cervical cancer [15, 19], and prostate carcinoma [20]. Thus, the involvement of PPARγ in TGZ-induced cytotoxicity seems to be cell type-dependent. Itami et al. previously reported that TGZ enhanced the luciferase activity of the PPAR response element (PPRE) in pancreatic cancer cell lines [9], indicating that TGZ certainly activated PPARγ to bind PPRE in these cells. Conversely, TGZ exhibited higher cytotoxicity than PGZ did (Fig. 1), although the affinity of TGZ to PPARγ is equal to that of PGZ [21]; this could be attributable to the differences in their chemical structure and partition coefficient, as well as their metabolism by the cells. Therefore, we concluded that the cytotoxic effect of TGZ in pancreatic cancer cells was independent of PPARγ activation.

To investigate the molecular mechanisms of TGZ-induced cell death, we first examined the involvement of apoptosis. TGZ cytotoxicity was accompanied by chromatin condensation (Fig. 3a), caspase-3 activation (Fig. 3b), and an increased Bax/Bcl-2 ratio (Fig. 3c) in MIA Paca2 cells, suggesting that TGZ induced cell death through apoptosis via the mitochondrial pathway. In PANC-1 cells, TGZ induced chromatin condensation more modestly than in MIA Paca2 cells, but did not elevate caspase-3 activity or increase the Bax/Bcl-2 ratio. Thus, other forms of death, such as caspase-independent apoptosis [14], autophagy, or necrosis, might participate to some extent in PANC-1 cells.

We next examined the involvement of cell growth, survival, and death signaling pathways. TGZ has been reported to regulate Akt and MAPK signaling [12, 17, 22–26]; therefore, we examined the phosphorylation of Akt and three classical MAPK proteins: ERK, JNK, and p38. Interestingly, Akt and ERK were activated by TGZ, although they are known to be involved in cell growth signaling (Fig. 4). Although Motomura et al. showed that TGZ reduced ERK signaling followed by inhibiting cell growth in pancreatic cancer cells [25], some groups reported opposite effects in other cancer cells, specifically that TGZ activated ERK and induced cell cycle arrest or cell death [19, 22, 24, 26]. The reason for these inconsistent results is unclear. In addition, we examined the effects of an ERK inhibitor, U0126, and an Akt inhibitor on TGZ-induced cell death, and we did not observe recovery of cell viability (data not shown). This suggests that the activation of ERK and Akt may be caused by PPARγ activation by TGZ, but that their signaling is unrelated to TGZ cytotoxicity in MIA Paca2 and PANC-1 cells. We then examined the involvement of other MAPK signaling proteins, which are known to mediate cell death signaling. TGZ activated p38 signaling, but co-exposure to a p38 inhibitor, SB202190, showed no effect on cell viability, suggesting that p38 signaling also did not contribute to TGZ-induced cell death (Fig. 4). However, we confirmed that TGZ activated JNK signaling, and a JNK inhibitor, SP600125, significantly mitigated the cytotoxic effects of TGZ in MIA Paca-2 cells (Fig. 4). Previous reports showed that the activation of JNK signaling induced mitochondria-mediated apoptosis through regulation of the Bcl-2 family [27, 28]. Taken together, the effects of TGZ in MIA Paca2 cells may very well occur through activation of JNK signaling and induction of apoptosis via reduction of Bcl-2 and activation of caspase-3. This is the first report to reveal the involvement of the JNK pathway in TGZ-mediated cytotoxicity in pancreatic cancer cell lines. However, the detailed mechanism in PANC-1 cells remains unclear. Several groups have revealed that TGZ caused G_1_ phase arrest through the up-regulation of p21 and p27, and caused the activation of autophagy in pancreatic cancer [9, 29, 30]. Moreover, various pleiotropic mechanisms have been reported, such as the regulation of p53, GADD [22, 31], β-catenin [32], and G_2_/M arrest [12, 31], indicating that TGZ-mediated cytotoxicity in cancer cells may be attributable to “off-target” mechanisms [33]. Some of these mechanisms could be involved in PANC-1 cells. It also remains unclear why the mechanisms of action differed between the studied cell lines, and further studies are required.

As metastasis is one of the most common problems in pancreatic cancer, we next investigated the effects of TGZ on cell invasion and cell migration. While TGZ significantly inhibited cell migration, the overall magnitude of inhibition was slight (Fig. 5). Additionally, there was no effect on cell invasion, suggesting that TGZ had little effect on pancreatic cancer metastasis. In our experiments, we used lower concentrations of TGZ and observed no effect on cell viability. Motomura et al. [34] have shown that TGZ suppressed cell invasion or cell migration, but their experimental conditions were different in terms of TGZ concentration, type of chemoattractant used, and analysis undertaken. Based on our findings, we suggest that TGZ is not a good candidate for the suppression of pancreatic cancer metastasis. However, more confirmatory data are needed.

Since the in vivo effects of TGZ against pancreatic cancer have not been evaluated thus far, we administered TGZ to mice inoculated with MIA Paca2 cells. Tumor growth was significantly inhibited by TGZ administration, and the body weights of mice did not change (Fig. 6), suggesting the absence of marked adverse effects. TGZ was withdrawn from market as an anti-diabetic drug because of severe liver toxicity [35]. On the other hand, existing non-oncological drugs could be useful for cancer therapy based on the notion of drug-repositioning [36]. Thus, TGZ could be an effective alternative for the treatment of pancreatic cancer. Moreover, combination therapy may be another useful strategy, as synergistic effects were reported with the combination of TGZ and chemotherapeutic agents [30, 37].

## Conclusions

A PPARγ agonist, TGZ, showed cytotoxicity in two pancreatic cancer cell lines in a PPARγ-independent manner. This study revealed that TGZ-mediated cytotoxicity occurred via the JNK pathway and mitochondria-mediated apoptosis, but our data also indicated the involvement of other types of cell death. In addition, we demonstrated, for the first time, the in vivo antitumor effects of TGZ in pancreatic cancer without marked adverse effects. Therefore, our study shows that TGZ administration might be a valuable approach for the treatment of pancreatic cancer, and TGZ warrants further investigation regarding its detailed mechanisms and clinical efficacy.

## Abbreviations

ERK: Extracellular signal-regulated kinase
JNK: c-Jun N-terminal kinase
MAPK: Mitogen-activated protein kinase
PGZ: Pioglitazone
PPAR: Peroxisome proliferator-activated receptor
PPRE: PPAR response element
TGZ: Troglitazone
